# Supplementary material for: Cure of experimental Trypanosoma vivax infection with a single dose of an unmodified antibody-based drug targeting the invariant flagellum cell surface protein IFX
Source: mBio. 2025 Oct 20;16(11):e02064-25. doi: 10.1128/mbio.02064-25 (PMC12607885; doi:10.1128/mbio.02064-25)
Supplement: Supplemental Figures — Fig. S1 and S2. [file mbio.02064-25-s0001.pdf]

Supplementary information

Autheman *et al*.

"Cure of experimental *Trypanosoma vivax* infection with a single dose of an unmodified antibody-based drug targeting the invariant flagellum cell surface protein IFX"

Supplementary Figure 1

>8E12_light_chain
MEFQTQVLMSLLLCMSGAAADIVLTQSPASLAVSLGQRATISCRASESVDKYGISFMNWFQQKPGQ
PPKLLIYAASNQGSGVPARFSGSGSGTDFSLNIHPMEEDDTAVYFCQQSKEVPYTFGGGTKLEIK*RA*
DAAPTVSIFPPSSEQLTSGGASVVCFLNNFYPKDINVKWKIDGSERQNGVLNSWTDQDSKDSTYSMS
*STLTLTKDEYERHNSYTCEATHKTSTSPIVKSFNRNEC**

>8E12_heavy_chain
MKLPVLLVVLLLFTSPASSSEVKIEESGGGLVQPGGSMKLSCDASGFTFSDAWMDWVRQSPEKGLE
WVAEIRNKADNHATYYAESVKGRFTISRDDSKSSVYLQMNSLRAEDTGIYYCTRTSFAYWGQGTLVT
VSA*ARPTAPSVYPLAPVCGDTTGSSVTLGCLVKGYFPEPVTLTWNSGSLSSGVHTFPAVLQSDLYTL*
SSSVTVTSSTWPSQSITCNVAHPASSTKVDKKIEPRGPTIKPCPPCKCPAPNLLGGPSVFIFPPKIKDV
LMISLSPIVTCVVVDVSEDDPDVQISWFVNNVEVHTAQTQTHREDYNSTLRVVSALPIQHQDWMSGK
EFKCKVNNKDLPAPIERTISKPKGSVRAPQVYVLPPPEEEMTKKQVTLTCMVTDFMPEDIYVEWTNN
*GKTELNYKNTEPVLDSDGSYFMYSKLRVEKKNWVERNSYSCSVVHEGLHNHHTTKSFSRTPGK**

Supplementary Figure 1. The protein sequences of the anti-IFX 8E12 monoclonal antibody light and heavy chains. Secretion signal peptides are shown in bold, and antibody constant regions in italics. The closest sequence similarity matching to mouse V and J region gene segments were: light chain *Igkv3-2*01* and *Igkj2*01*; heavy chain *Ighv6-6*01* and *Ighj3*01*. Complementarity determining regions in both antibodies are underlined.

Supplementary Figure 2

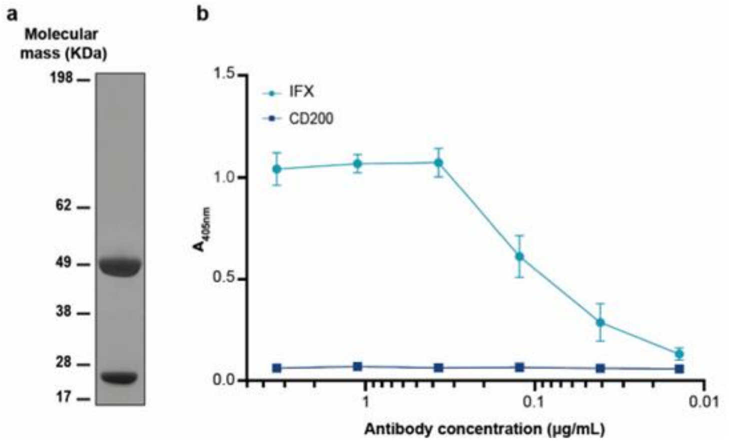

Supplementary Figure 2. Preparation of a pure and active preparation of the 8E12-IgG2a-formatted monoclonal antibody. HEK293-6E cells were transfected with a single plasmid encoding both light and heavy chains of the 8E12 monoclonal antibody and the expressed antibody purified using protein G resin. (**a**) One microgram of purified antibody was resolved on an SDS-PAGE gel under reducing conditions and shown to be highly pure. (**b**) Purified 8E12 antibody is highly active. Purified 8E12 antibody was serially diluted and tested for binding activity to the entire ectodomain of IFX expressed as a soluble enzymatically monobiotinylated recombinant protein captured on a streptavidin-coated microtitre plate; the ectodomain of human CD200 was used a negative control. Data points represent means ± SD; *n*=4.
